# CT-Guided Percutaneous Radiofrequency Thermocoagulation for Recurrent Trigeminal Neuralgia After Microvascular Decompression: A Cohort Study

**DOI:** 10.1097/MD.0000000000001176

**Published:** 2015-08-14

**Authors:** Guang-Hui Lai, Yuan-Zhang Tang, Xiao-Ping Wang, Hong-Jun Qin, Jia-Xiang Ni

**Affiliations:** From the Department of Pain Management (G-HL, Y-ZT, X-PW, J-XN), Xuanwu Hospital, Capital Medical University; and Department of Radiology (H-JQ), Beijing Electric Power Hospital, Beijing, China.

## Abstract

This article evaluates the long-term outcomes of computed tomography (CT)-guided percutaneous radiofrequency thermocoagulation (PRT) for patients with recurrent trigeminal neuralgia (TN) after microvascular decompression (MVD).

This is a retrospective study of 41 patients with intractable TN who after MVD underwent CT-guided PRT procedures between 2002 and 2012.

The mean length of follow-up after PRT was 44.4 months. Immediate pain relief was in 37 patients (90.2%); the percentage of patients who remained in “excellent” or “good” pain relief condition after CT-guided PRT procedure was 85% at 1 year, 80% at 2 years, 51% at 5 years, and 41% at 10 years. Six patients received the second PRT and all achieved “excellent” or “good” pain relief. In total, 34 of these patients (82.9%) received multi-PRT procedure and remained satisfied with their pain relief during the follow-up period. Postoperative complications included facial numbness in 36 patients, limited eyes opening in 1 patient, ear paresthesia in 1 patient, no tears in 1 patient, and taste hypesthesia in 1 patient; these symptoms were all improved in the process of follow-up and their life had not severely affected. No mortality was observed during and after CT-guided PRT procedures.

CT-guided PRT should be considered as an alternative treatment for patients with recurrent TN after MVD.

## INTRODUCTION

Trigeminal neuralgia (TN) is a severe syndrome characterized by facial pain that is described as excruciating and causes serious impairments of the quality of life. Since Jannetta^1,2^ improved and generalized the microvascular decompression (MVD), it was considered as the first choice for TN patients. The biggest advantage of MVD, which remove the vascular compression of root entrance of the trigeminal nerve, was maintaining the normal facial sense with long-term effective pain relief and avoiding the appearance of the facial numbness and discomfort after procedure. However, some patients can only get partial pain remission or even be invalid, the reported annual recurrence rate range from 1% to 5%.^3–7^ How to cure these refractory TN patients is challenging for TN treatment.

CT-guided percutaneous radiofrequency thermocoagulation (PRT) of the trigeminal gasserian ganglion, an effective and less-invasive treatment, has gained wide acceptance in the treatment of TN patients who are refractory to medical therapy. This technique has been popularized since 1974^8^ and now PRT is the most commonly performed ablative procedure at the level of the gasserian ganglion for TN patients.^9^ The advantage of PRT is that it is safely used for multirepeated procedure. Repeated PRT provides long-term pain relief benefits to patients with recurrent TN after single PRT has been reported^10,11^; however, little is known regarding clinical outcome analysis solely for patients with recurrent TN following MVD procedures.

In our unit, CT-guided PRT has become the treatment of choice for refractory TN since 2002, and there were several studies focused on evaluating the long-term outcomes of PRT for the patients with TN.^11,12^ The present study aimed to evaluate the clinical outcomes of CT-guided PRT procedure in patients with TN after MVD.

## MATERIAL AND METHODS

From January 2002 to December 2012, 996 patients underwent CT-guided PRT procedure for classic TN at the Department of Pain Management in Xuanwu Hospital, Beijing, China; 41 patients of them who had received MVD previously were included in the final analysis.

This study was approved by the Ethical Committee of the Xuanwu Hospital. Prior to the CT-guided PRT procedure, all patients were informed about the procedure and its possible complications, and written informed consent was obtained. Classic TN was diagnosed according to the International Classification of Headache Disorders II (2004) criteria.^13^ Follow-up was conducted via telephone interview and medical records.

### PRT Procedures

CT-guided PRT procedure was performed according to our previously reported studies.^12,14^ The patient was taken to our disinfected CT examination room and was placed in a supine position with their head overhanging on the CT scanner bed. Each patient's vital signs were monitored during the entire procedure. The puncture of gasserian ganglion was according to Hartel anterior route. The best puncture approach to the oval foreman and the corresponding skin insertion point was determined by CT scanning. After sterilization, the insertion point was anesthesized with 1% lidocaine. Then, a 22-gauge radiofrequency-insulated needle with a 5-mm active tip was inserted. The insertion angle and the advanced depth to the foramen ovale of the needle were according to the best puncture approach. While piercing the needle into the foramen ovale, repeated CT scan was used to reconfirm the position of the needle tip. After verification, motor (2 Hz, 1 ms) and sensory (50 Hz, 0.1 ms) were performed to confirm or readjust the needle tip position to confirm the accuracy. After certifying the proper location, the patient was administered intravenous anesthesia with propofol (1–2 mg/kg) and supplemented with facemask oxygen. No tracheal intubation was performed. The gasserian ganglion was thermally coagulated with radiofrequency at 75°C for 120 seconds.

### Pain Assessment

Pain degree was evaluated at the baseline and postoperative follow-up, using the Barrow Neurological Institute (BNI) grading score.^15^ The outcomes were classified as follows: “excellent” results were defined as BNI grade I (no pain and no medicine), “good” results were defined as BNI grade II (occasional pain, no medicine) or BNI grade III (moderate pain, controlled with medicine), and “poor” results were accomplished when the pain remained at an intensity BNI ≥ grade IV (moderate or severe pain, not controlled by medicine) after treatment; CT-guided PRT were repeated in cases of BNI grade IV or V after the first PRT procedure.

### Statistic

The data were analyzed using the statistical package for the social sciences version 17.0 (SPSS, Chicago, IL). Kaplan–Meier curves were calculated to determine the percentage of patients that were in the “excellent” and “good” outcome category after first CT-guided PRT. Patient follow-up was censored at last contact (n = 20) and time of subsequent surgery (n = 6).

## RESULTS

Table 1 shows detailed background information of all 41 patients, including patient age, sex, and original location of pain. Their median age was 63.0 years (range 25–79). The mean follow-up period was 44.4 months (range 13–102). The overall mean time after MVD to recurrence was 14.4 months (range 2–120). No patients lost to follow-up.

**TABLE 1:**
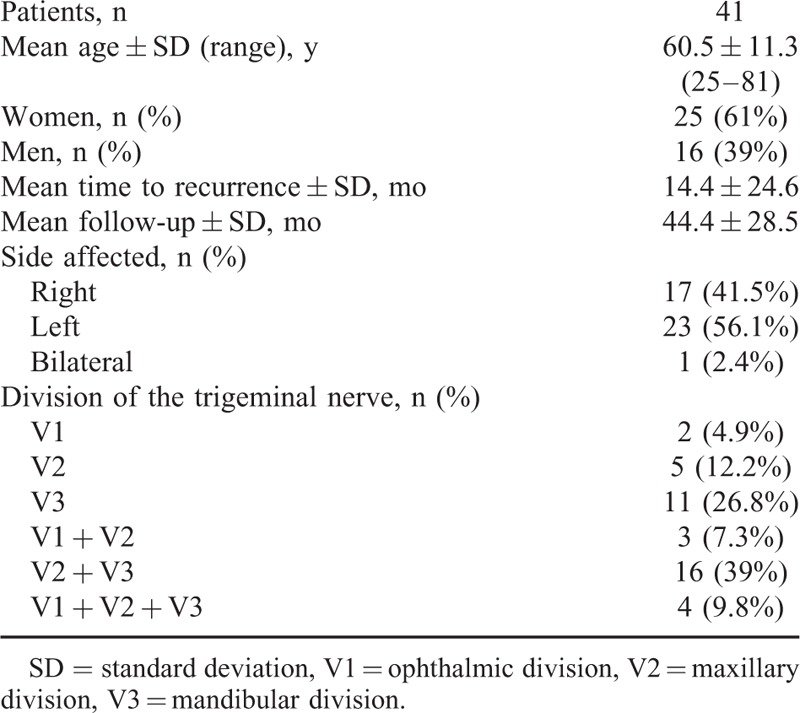
Patients’ Characteristic

**FIGURE 1:**
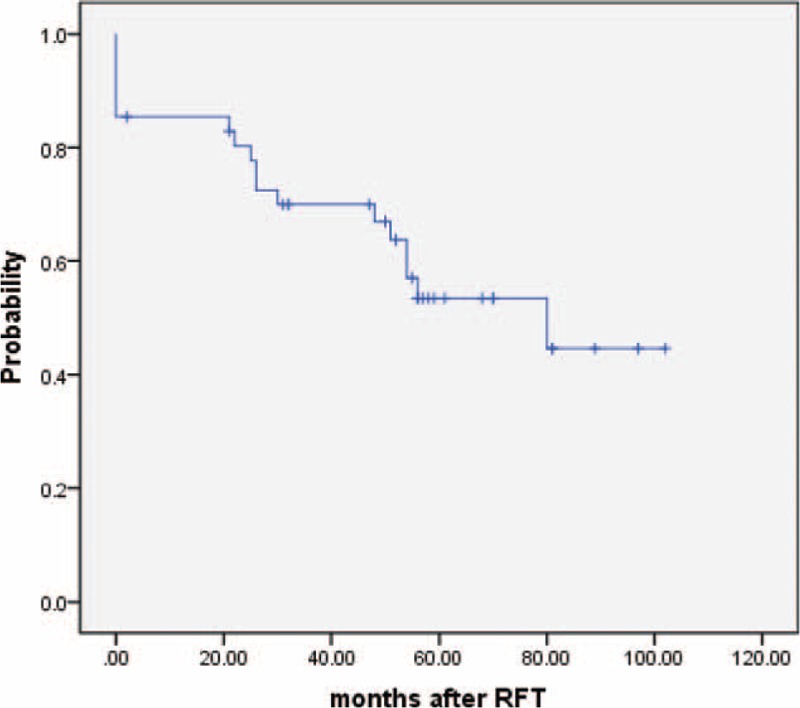
Kaplan–Meier curves of “excellent” or “good” results after percutaneous radiofrequency thermocoagulation (PRT) for recurrent trigeminal neuralgia after microvascular decompression (MVD).

After the first PRT, 37 patients (90.2%) achieved immediate pain relief and 4 patients (9.8%) experienced recalcitrant symptoms. The Kaplan–Meier curve for “excellent” or “good” outcomes of 41 patients after first CT-guided PRT procedures are shown in Figure 1, in which the event was set for failure or recurrence; the percentage of patients with “excellent” or “good” outcomes (pain intensity ≤ BNI grade III) was 85% at 1 year, 80% at 2 years, 51% at 5 years, and 41% at 10 years.

Of the 37 patients with immediate pain relief, 23 patients (62.2%) maintained pain relief with “excellent” results for the duration of their follow-up (mean 43.4 months, range 13–87 months) and their medication were tapered off a period of several weeks; 5 patients (13.5%) achieved “good” results and got effective pain control with drugs (mean 48.6 months, range 8–42 months). The remaining 9 patients (24.3%) experienced pain recurrence with “poor” results (mean 38.3 months, range 2–47 months); 4 of them underwent stereotactic radiosurgery including 2 patients who achieved “excellent” pain relief with a follow-up of 12 and 27 months, respectively. Another 2 patients did not achieve pain relief; 5 of 9 patients with “poor ” results after first PRT underwent second procedure. After the second PRT, “excellent” pain relief was obtained in 1 patient when checked at 15 months of follow-up; 2 patients had “good” pain relief result switched to drug treatments, which resulted in an effective pain control when assessed 17 months later; 2 patients were with “poor” results after the second procedure at 10 months, 1 received a third PRT procedure and the other underwent stereotactic radiosurgery and did not achieve pain relief.

Of 4 patients whose pain had no relief after first PRT, 2 of them experienced second PRT, and his pain had relieved completely and maintained with follow-up of 18 and 27 months, respectively. The other 2 patients both underwent stereotactic radiosurgery, 1 of them got partial pain relief and needed drug to control pain attack with follow-up of 31 months, and the other patient was not still relieved and he died in myocardial infarction (which was not related to TN surgical procedure) 24 months after stereotactic radiosurgery.

## COMPLICATIONS

Thirty-six of all patients (87.8%) had a mild-to-moderate degree of facial numbness after PRT. This sense gradually alleviated in follow-up. No painful dysesthesia occurred after PRT. Additional postoperative complications included limited eyes opening in 1 patient, paresthesia of ipsilateral ear in 1 patient, no tears in 1 patient, and taste hypesthesia in 1 patient; these symptoms were improved in the process of postoperative follow-up and their life had not been affected. There was no mortality and no permanent cranial nerve deficit except dysesthesia observed in these 41 patients.

## DISCUSSION

Given that most cases of TN are believed to be caused by “neurovascular compression,” MVD was thought to be the preferred method for TN treatment. But approximately 15% of patients may not have a significant vascular compression, or adequate decompression may not be achieved safely in them.^16^ Devor et al^17^ believed that compression and demyelination of sensory nerve is generally associated with tough numbness or vibration sense, and not pain. Additionally, MVD procedure has certain recurrence rates. Cho et al^18^ reported that in the first year, the recurrence rate was 14% with 2% to 3.5% growth per year, and >25% after 5 years. Some studies revealed that the pain did not ease completely after MVD; insufficient decompression, without significant vascular compression, or vascular compressed the nerve again might be reasons, but they were not verified,^19,20^ at the same time long history (>8 years) is also a negative factor.^21^ There were lots of report about reoperation after MVD,^22–26^ but recurrent TN after first MVD poses a management challenge because of arachnoid adhesions and abnormal anatomical relationships,^22^ and complications are relatively higher than first MVD.^26,27^ A large-scale MVD study reported that the morbidity rate of MVD is about 0.3%.^28^ The effectiveness of MVD in the unobvious vessel compression group was worse than those in the obvious group.^29^ Meanwhile, patients would become elder when it recurred that lead to higher risks of operation and endotracheal anesthesia. More minimally invasive procedures are needed for these patients.

No need for endotracheal anesthesia and short hospitalization make PRT one of the most common procedures for TN. This procedure is especially suitable for elderly, and patients with poor fitness or who refuse to perform MVD. A nationwide study of 3 invasive treatments for TN found that after MVD, most patients had a PRT as second procedure.^30^ However, PRT and MVD have similar initial success rate and recurrence rate; a lower rate of adverse events makes it more acceptable, so neurosurgeons should be familiar with both the techniques to select the best treatment for each patient.^16,31^

In our study, 41 patients who had “poor” outcomes after MVD received CT-guided PRT procedure, 37 (90.2%) patients achieved immediate pain relief, and 34 (82.9%) received multi-PRT procedure and remained satisfied with their pain relief during the follow-up period. Neither mortality nor life-threatening complications was observed, and the rate of adverse events conformed to the previous reports.^10,32^ Our results suggest that PRT is a safe and effective therapy for the patients with recurrent TN after MVD. PRT management of TN is based on interrupting the pain by partial damage to the trigeminal nerve fiber; the destruction of the surrounding tissue is very limited and repeated PRT could be safe and effective.^11^

The success rates of initial MVD, repeated MVD, repeated PRT, and PRT after MVD are shown in Table 2. The success rate is still high, although comparatively lower than repeated MVD; however, the data of success rate at 3 and 5 years of repeated MVD is deficit. Six patients received “excellent” or “good” result after repeated PRT in our study. In total, 34 (82.9%) patients who underwent multi-PRT obtained excellent or good pain relief. So PRT is the procedure of choice for recurrent TN after MVD, because its results are comparable to those of MVD, especially for patients with poor fitness or who refuse to receive craniotomy surgery again.

**TABLE 2:**
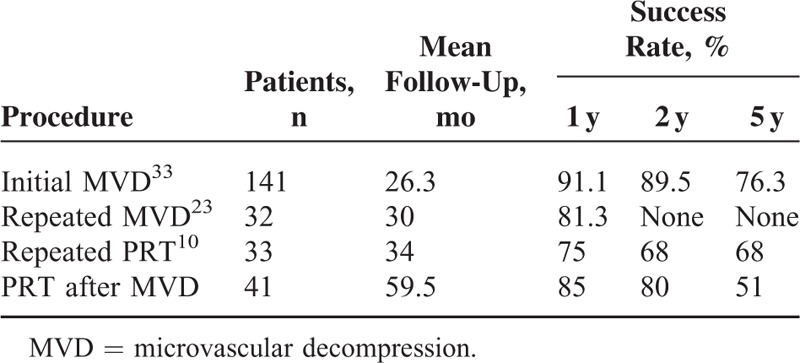
Comparison of Success Rates of Different Procedures

The most common complication is facial numbness; in our study, 36 patients (87.8%) experienced significant facial numbness. Achievement of numbness is a prerequisite for prolonged pain cessation of PRT procedure.^12^ Fortunately, facial numbness of all patients after PRT is mild, limited, and well tolerated by most patients; no painful dysesthesia occurred.

Four patients who underwent MVD, PRT, and stereotactic radiosurgery did not achieve successful pain relief, as we inferred in the previous study; there might be a subpopulation of TN patients resistant to all currently available treatment modalities and this remains unclear which patient might fail to benefit from PRT. Neural plasticity up to the gasserian ganglion may be dominant for this refractory pain, and these patients might benefit from motor cortex stimulation or deep brain stimulation. These refractory patients should be further studied in future.

## CONCLUSION

In the present study, data from 41 patients with recurrent TN after MVD who underwent CT-guided PRT procedure are presented. PRT procedure may achieve long-term pain relief with minimal rate of complications in patients with recurrent TN after MVD. Given that CT-guided PRT is a safe and effective treatment procedure to perform with a minimal complication rate, it should be considered a practical treatment option in treating recurrent TN after MVD, especially for patients with poor fitness or who refuse to receive craniotomy surgery again.
